# Safety, Tolerability, and Pharmacokinetics of EGT710, a Novel Coronavirus M^pro^ Inhibitor, in Healthy Adults: A Randomized, Single‐ and Multiple‐Dose Study

**DOI:** 10.1002/cpdd.70115

**Published:** 2026-10-24

**Authors:** Jart A. F. Oosterhaven, Jonathan Trolander, Lidiya Bebrevska, Hilmar Schiller, Begum Alaybeyoglu, Suzanne Gaudet, Allison Claas, Kamal Kumar Balavenkatraman, Renee Bergeron, Fernanda V. Duraes, Frederique Chaperon, Swati Ghanshani, Elisabete Goncalves, Steven J. Kovacs

**Affiliations:** ^1^ ICON Plc Groningen The Netherlands; ^2^ CA CSI CVM, Biomedical Research, Novartis Cambridge MA USA; ^3^ Global Health, Research & Development, Pharmacokinetics, Novartis Pharma AG Basel Switzerland; ^4^ Early Development Analytics, Development, Novartis Pharmaceuticals Corporation Cambridge MA USA; ^5^ Preclinical Safety Biomedical Research, Novartis Pharma AG Basel Switzerland; ^6^ Preclinical Safety Novartis Pharmaceuticals Corporation East Hanover NJ USA; ^7^ CS&I Immunology, Biomedical Research Novartis Pharma AG Basel Switzerland; ^8^ Biostatistics & Pharmacometrics Novartis Healthcare Private Limited Mumbai India; ^9^ Translational Medicine Novartis Pharmaceuticals Corporation East Hanover NJ USA

**Keywords:** bioavailability, coronavirus, COVID‐19, EGT710, M^pro^ inhibitor, pharmacokinetics

## Abstract

Antiviral therapy is needed to address acute coronavirus disease. EGT710 is a novel low‐molecular‐weight inhibitor of the coronavirus main protease (M^pro^). This first‐in‐human study evaluated the safety, tolerability, and pharmacokinetics of EGT710 in healthy adults. The study conducted in the Netherlands involved three parts: a blinded, placebo‐controlled, 3:1 randomized, single ascending dose Part A (n = 39); a multiple ascending dose Part B (n = 31); and an open‐label, three‐period, six sequence, randomized, cross‐over Part C (n = 18) to evaluate food and formulation effects on EGT710 bioavailability. EGT710 was well tolerated. All adverse events (AEs) were mild (Grade 1), with no serious AEs or discontinuations. Aside from mild gastrointestinal AEs associated with EGT710, AE frequency was similar between groups with no dose‐dependent or dose‐limiting adverse effects. EGT710 was rapidly absorbed (median T_max_ 2.0 h), showed sub‐proportional AUC and C_max_ increases and no significant accumulation with daily dosing. Bioavailability was comparable between suspension and capsule formulations. Food increased C_max_ by 20% and AUC by 27%. EGT710 700 mg once daily for 7 days modestly induced CYP3A4 activity but had no effect on organic anion transporting polypeptide (OAT1/3) activity. Oral EGT710 (capsule and suspension) was safe and well tolerated, with pharmacokinetics that support further development as a treatment for coronavirus disease.

Coronavirus disease has and will continue to pose notable public health challenges and economic instability worldwide.^1^, ^2^ From the recent coronavirus disease 2019 (COVID‐19) pandemic to the earlier epidemics of the severe acute respiratory syndrome (SARS) in 2003 and Middle East respiratory syndrome (MERS) in 2012, the morbidity and mortality associated with acute coronavirus disease are considerable.^3^, ^4^, ^5^ As of February 2026, approximately 779 million cases of COVID‐19 have been reported worldwide, including 7.1 million associated deaths.^6^

Coronaviruses are enveloped, positive‐stranded, ribonucleic acid (RNA) viruses that evolve (mutate) rapidly and have a wide range of natural animal hosts, making zoonotic transmission and the emergence of new outbreaks inevitable.^1^, ^7^ SARS‐CoV, SARS‐CoV‐2, and MERS‐CoV, which are the causative agents of SARS, COVID‐19, and MERS, respectively, are known to cause gastrointestinal and respiratory infections in humans and animals, resulting in mild to severe disease involving multiple organs, especially the respiratory system.^2^, ^3^, ^8^, ^9^

Vaccines were the first novel therapeutic intervention to address the COVID‐19 pandemic, providing protection against severe illness, hospitalization, and death.^10^ However, vaccine‐induced and natural immunity may not confer protection against future infections with novel coronavirus strains. Broadly efficacious and direct‐acting antiviral medications are crucial for treatment of acute coronavirus disease, especially for those at high risk of severe or progressive disease.^11^ Targeting SARS‐CoV‐2's functional proteins, such as its main protease (M^pro^), has been a primary therapeutic strategy in developing antiviral therapies. M^pro^ cleaves viral polyproteins, which are essential for viral replication and transcription. Coronavirus M^pro^ has no known homologs in the human genome, making it a promising target for drug development.^8^, ^9^

Nirmatrelvir, the first oral M^pro^ inhibitor, was approved by the US Food and Drug Administration on May 25, 2023, for co‐administration with ritonavir (as Paxlovid) for the treatment of mild‐to‐moderate COVID‐19 in adults at high risk of progression to severe COVID‐19.^12^ The COVID‐19 treatment guidelines panel endorse ritonavir‐boosted nirmatrelvir as the preferred treatment, when necessary, for the treatment of non‐hospitalized adults.^11^ Nevertheless, continuous emergence of SARS‐CoV‐2 variants, growing vaccine hesitancy, and the risk of drug–drug interactions with ritonavir‐boosted nirmatrelvir necessitate the development of alternative therapeutic options that offer similar or superior efficacy, safety, and tolerability.

EGT710 is a novel low‐molecular‐weight M^pro^ inhibitor with demonstrated antiviral activity against the known human coronaviruses in non‐clinical in vitro and in vivo experiments.^13^ In biochemical assays, EGT710 is active against M^pro^ from the four genera of coronaviruses, including the SARS‐CoV‐2 variants (USA‐WA1/2020, alpha/B.1.1.7, beta/B.1.351, delta/b.1.617.2, and omicron/BA.1) as well as other human coronaviruses in cellular assays. EGT710 also demonstrated efficacy in a mouse model of SARS‐CoV‐2 infection, with a twice‐daily dose of 10 mg/kg that reduced viral titers by >5 logs.^13^

Here, we report the results of a first‐in‐human study (EudraCT 2022‐001905‐37) that evaluated the safety, tolerability, and pharmacokinetics (PK) of EGT710 following oral administration of two different formulations to healthy adults. The study also investigated the impact of food on EGT710 absorption and the effect of EGT710 on endogenous markers of CYP3A4 activity and organic anion transporting polypeptide (OAT1/3) activity.

## Methods

### Study Design

This was a randomized study consisting of three parts: blinded and placebo‐controlled Parts A and B, and open‐label Part C. The study was conducted between October 2022 and September 2023 at a single center in the Netherlands. The study treatments were capsules, powder for oral suspension (referred to as “suspension” hereafter), and their matching placebos. Part A was a 3:1 randomized, participant‐ and investigator‐blinded, placebo‐controlled, single ascending dose (SAD) design conducted in five separate cohorts: EGT710 80, 240, 720, and 1100 mg as capsules and 300 mg as suspension or matching placebo. Part B was a 3:1 randomized, participant‐ and investigator‐blinded, placebo‐controlled, multiple ascending dose (MAD) design, conducted in four separate cohorts: EGT710 300, 460, and 700 mg as capsules and 700 mg as suspension or matching placebo once daily for 7 days. Part C was an open‐label, randomized, three‐period, six‐sequence, crossover design evaluating the effect of food (FE) on the PK of EGT710 600 mg and the bioavailability of the suspension formulation relative to the capsule formulation after single‐dose administration (rBA). The durations for Parts A, B, and C of the study were 31± 2 days, 37 ± 2 days, and 41 ± 2 days, respectively, including the screening period (Figure 1).

**Figure 1:**
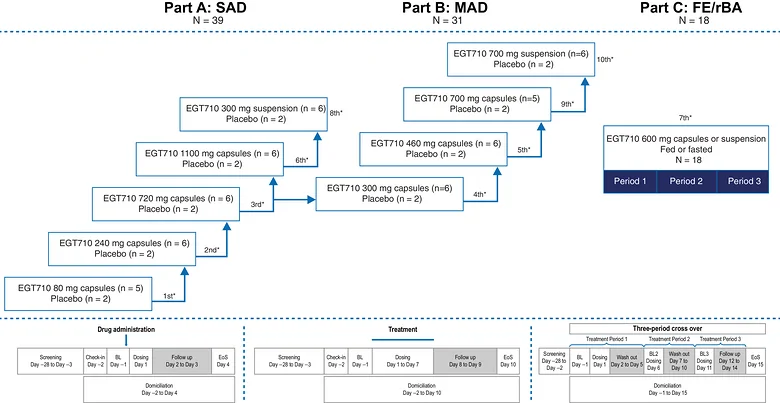
Study design. BL, baseline; EoS, end of study; FE/rBA, food effect/relative bioavailability; MAD, multiple ascending dose; SAD, single ascending dose. ^*^Cohorts were completed in the following order: SAD 1 (80 mg), SAD 2 (240 mg), SAD 3 (720 mg), MAD 1 (300 mg), MAD 2 (460 mg), SAD 4 (1100 mg), FE/rBA (600 mg), SAD 5 (300 mg), MAD 3, and MAD 4 (700 mg).

### Treatment

The treatment administered involved two different formulations of EGT710, namely a capsule and a suspension, both with matching placebo. The placebo was administered as the same formulation as that of EGT710 in all dosing cohorts of Parts A and B. Part C featured three conditions with single‐dose administration: capsule (reference formulation) and suspension (test formulation) in a fasted state, and suspension (test formulation) in a fed state. Participants received three single doses of EGT710 on Days 1, 6, and 11, separated by washout periods, with end‐of‐study (EoS) assessments on Day 15 (Figure 1). For the food effect portion, participants received a breakfast meal within 30 min of dosing which consisted of two eggs fried in ∼15 mg of butter/margarine (∼100 g), two slices of bread with ∼15 mg of butter/margarine, one portion of bacon (∼40 g), one portion of fried potatoes (∼115 g), and one glass of whole milk (∼240 mL).

The starting dose of EGT710 was 80 mg based on preclinical safety data. For subsequent cohorts in Parts A and B, including doses of the suspension cohorts after completion of Part C, dose levels and escalation decisions were guided by reviews of safety and PK data, including PK modeling and simulation. The dose increments did not exceed the preceding dose by more than three‐fold.

### Study Population

Male and female adults aged 18–65 years with a body mass index of ≤35 kg/m^2^ who were judged to be healthy by medical history, physical examination, vital signs, laboratory tests, and electrocardiogram (ECG) were eligible to participate. Study participants should not have had any chronic illness. Female participants had to be naturally or surgically incapable of becoming pregnant. Sexually active males had to agree to use condoms to prevent pregnancy and/or inadvertent exposure of partners to study treatment through seminal fluid. Use of other investigational drugs at the time of screening, or within five half‐lives or 30 days of screening, whichever was longer, was prohibited. Participants with a positive SARS‐CoV‐2 test result at check‐in or baseline were excluded. Participants who met the eligibility criteria at screening (Days −28 to −3) were admitted to the study site on Day −2, underwent baseline evaluations on Day −1, and remained domiciled until completion of the EOS evaluations for each part of the study.

Use of concomitant therapy (prescription or non‐prescription) was not permitted within 2 weeks before initial dosing and until all study evaluations had been performed. This included herbal, dietary, fitness, bodybuilding, athletic performance‐enhancing supplements, and vaccinations. However, standard daily vitamins taken up to 48 h before domiciliation and medications required to treat adverse events (AEs) were permitted.

### Study Objectives and Assessments

The primary objective was to assess the safety and tolerability of EGT710 following oral administration. This included assessment of AEs, vital signs, laboratory tests, and ECG. AEs were evaluated using the Medical Dictionary for Regulatory Activities (MedDRA), and AE intensity was graded according to Common Terminology Criteria for Adverse Events (CTCAE) version 5.0 or higher.

Secondary objectives included evaluation of the PK of EGT710 following the administration of single and multiple oral doses, estimation of the rBA of EGT710 following single‐dose administration of the suspension (test formulation) relative to the capsules (reference formulation) under fasting conditions, and evaluation of the impact of food on the PK of EGT710 following single‐dose administration of the capsules.

PK parameters were assessed in all participants who received at least one dose of EGT710. For Parts A and C (i.e., single‐dose administration), blood samples were collected prior to dose administration, every 0.5 h until 2.5 h post‐dose, and then at 4, 5, 6, 8, 12, and 24 h, and, finally, every 12 or 24 h until Day 4 (EoS). In Part B (i.e., daily dosing for seven consecutive days), the samples were collected prior to dose administration and every 0.5 h until 2.5 h, followed by 4, 5, 6, 8, 12, and 24 h after the first and seventh doses. On Days 2–7, samples were collected before drug administration. Finally, samples were collected every 24 h after the last dose until Day 10 (EoS).

EGT710 concentrations were measured using validated liquid chromatography–tandem mass spectrometry with a lower limit of quantification of 5 ng/mL. PK parameters were determined using Phoenix WinNonlin (Version 8.3.4 or higher). Renal clearance and the amount of drug excreted in the urine were assessed in 0–24 h and 24–72 h pooled urine collections after the last dose on Day 7.

Exploratory objectives included assessment of the effect of EGT710 on endogenous markers of CYP3A4 and OAT activity. The ratio of 6β‐hydroxycortisol to cortisol in urine was used as the marker for CYP3A4 activity^14^, ^15^ and was assessed in pooled urine during Part B on Day −1 (0–4, 4–12, and 12–24 h), Day 7 (0–4, 4–12, and 12–24 h), and Day 9 (48–52, 52–60, and 60–72 h). Plasma pyridoxic acid was used as the marker for OAT activity.^16^, ^17^ Pyridoxic acid in plasma was assessed on Day 1; Day 7 at −1, 0.5, 1.5, 2, 2.5, 4, and 8 h; and Day 8. These assessments were completed for Part B of the study only, and the analysis included only participants assigned to receive EGT710.

The acceptability and palatability of the suspension formulation were assessed using a paper questionnaire designed to evaluate the taste, after taste, and smell of the formulation. Participants were first asked if they noticed a taste, after taste, or smell. If participants responded “no,” then no further questions about that category were asked. If participants responded “yes,” they were asked to rate the taste, after taste, or smell as very good, good, bad, or very bad. This assessment was conducted by clinical staff for all participants in Part C during the dosing visit, in fed and fasted states.

### Statistical Analyses

Safety and tolerability variables were summarized descriptively by dose level in each study part. Exposure parameters (e.g., area under the curve [AUC], C_max_, elimination half‐life) were summarized by geometric (geo) mean (geo‐coefficient of variation [CV]%) while the time to maximum plasma concentration (t_max_) was summarized as median and range. Dose proportionality analyses for AUC and C_max_ from the SAD and MAD parts were performed only for cohorts receiving the capsule formulation because there was only one cohort in each part that received the suspension.

For Parts A and B, eight participants in each cohort were planned, of which six (minimum of five) received EGT710 and two received placebo per dose group. This approach was considered sufficient for safety and tolerability assessments and ensured >80% probability for an AE with an underlying occurrence rate of ≥24% to be observed at least once in the given cohort. For Part C, 18 participants were enrolled such that the 90% confidence interval (CI) for the PK parameter ratio falls within the range of 85%–117% of the observed geo‐mean ratio, with an assumed intra‐participant CV of 30%.

The data collected from the acceptability and palatability questionnaire for suspension formulation in Part C were listed by the corresponding treatment group, participant, and visit/time and summarized by treatment group and overall.

### Ethical Considerations

All participants in the study provided written informed consent. The study was conducted under the supervision and approval of an independent ethics committee, Stichting Beoordeling Ethiek Biomedisch Onderzoek, the Netherlands (EC approval code NOV22142‐22142X), adhering to the ICH E6 Guidelines for Good Clinical Practice, and the ethical principles of the Declaration of Helsinki.

## Results

A total of 88 participants were enrolled: 39 in Part A, 31 in Part B, and 18 in Part C. All enrolled participants completed the study (Figure S1). Most participants were White (87.5%) and male (95.5%). Baseline characteristics were comparable across cohorts (Table 1).

**Table 1:** Baseline Demographics

	Part A (SAD)							Part B (MAD)						Part C (FE/rBA)
	EGT710 Capsule					EGT710 Suspension		EGT710 Capsule				EGT710 Suspension		Capsule/Suspension
Characteristic	80 mg n = 5	240 mg n = 6	720 mg n = 6	1100 mg n = 6	Placebo n = 8	300 mg n = 6	Placebo n = 2	300 mg n = 6	460 mg n = 6	700 mg n = 5	Placebo n = 6	700 mg n = 6	Placebo n = 2	600 mg n = 18
Age (years), mean (SD)	28.4 (17.17)	29.3 (11.38)	23.0 (3.79)	27.5 (5.09)	33.9 (17.82)	25.2 (8.61)	25.5 (9.19)	46.2 (17.10)	42.0 (13.34)	39.8 (12.19)	28.7 (9.40)	36.7 (20.48)	37.5 (26.16)	30.4 (8.90)
Gender, n (%) Male Female	5 (100) 0	6 (100) 0	6 (100) 0	6 (100) 0	7 (87.5) 1 (12.5)	6 (100) 0	2 (100) 0	5 (83.3) 1 (16.7)	5 (83.3) 1 (16.7)	5 (100) 0	6 (100) 0	6 (100) 0	2 (100) 0	17 (94.4) 1 (5.6)
Race, n (%) White Black Asian Multiple	5 (100) 0 0 0	5 (83.3) 0 1 (16.7) 0	6 (100) 0 0 0	5 (83.3) 0 0 1 (16.7)	7 (87.5) 0 1 (12.5) 0	5 (83.3) 0 1 (16.7) 0	2 (100) 0 0 0	6 (100) 0 0 0	5 (83.3) 0 0 1 (16.7)	4 (80.0) 0 0 1 (20.0)	5 (83.3) 1 (16.7) 0 0	5 (83.3) 0 0 1 (16.7)	2 (100) 0 0 0	15 (83.3) 2 (11.1) 1 (5.6) 0
Ethnicity, n (%) Hispanic Not Hispanic	0 5 (100)	0 6 (100)	0 6 (100)	0 6 (100)	0 8 (100)	0 6 (100)	0 2 (100)	0 6 (100)	0 6 (100)	0 5 (100)	1 (16.7) 5 (83.3)	1 (16.7) 5 (83.3)	0 2 (100)	0 18 (100)
BMI at screening (kg/m^2^), mean (SD)	24.37 (2.61)	23.84 (3.39)	22.30 (1.22)	23.71 (3.97)	24.08 (2.78)	22.76 (2.33)	26.21 (4.70)	25.45 (5.79)	24.42 (1.99)	23.57 (3.18)	23.35 (2.42)	24.23 (4.60)	24.26 (0.02)	26.69 (3.53)

BMI, body mass index; FE/rBA, food effect/relative bioavailability; MAD, multiple ascending dose; SAD, single ascending dose.

The analysis set includes all the participants randomized into the active treatment and placebo groups.

### Safety and Tolerability

Overall, AE rates were similar between the treatment and placebo groups, with 35 of the 70 (50%) participants who received EGT710 and 9 of the 18 (50%) participants who received placebo, reporting at least one AE. In Part A, 8 of the 29 (27.6%) participants who received EGT710 and 4 of the 10 (40%) participants who received placebo reported at least one AE; in Part B, 15 of the 23 participants (65.2%) receiving EGT710 and 5 of the 8 participants (62.5%) receiving placebo reported ≥1 AE; in Part C, 12 of the 18 (66.7%) participants reported at least one AE. General and administration site conditions, mainly skin irritation related to medical devices (at sites of placement of the ECG, Holter leads, and intravenous cannulas), were the most common AEs across all parts of the study. Other common AEs included gastrointestinal disorders and nervous system disorders (Table 2).

**Table 2:** AEs Regardless of the Relationship with the Study Drug

*System Organ Class* Preferred Term, n (%)	EGT710 SAD (Part A)						
	Capsule					Suspension	
	80 mg n = 5	240 mg n = 6	720 mg n = 6	1100 mg n = 6	Placebo n = 8	300 mg n = 6	Placebo n = 2
**Participants with ≥1 AE**	**0**	**3 (50.0)**	**1 (16.7)**	**2 (33.3)**	**2 (25.0)**	**2 (33.3)**	**2 (100)**
***Nervous system disorders***	**0**	**3 (50.0)**	**1 (16.7)**	**1 (16.7)**	**0**	**1 (16.7)**	**0**
Dizziness	0	0	0	1 (16.7)	0	0	0
Headache	0	2 (33.3)	0	0	0	1 (16.7)	0
Somnolence	0	1 (16.7)	1 (16.7)	0	0	0	0
***General disorders and administration site conditions***	**0**	**0**	**1 (16.7)**	**1 (16.7)**	**1 (12.5)**	**0**	**0**
Medical device site irritation	0	0	1 (16.7)	1 (16.7)	1 (12.5)	0	0
***Musculoskeletal and connective tissue disorders***	**0**	**0**	**0**	**0**	**0**	**1 (16.7)**	**2 (100)**
Myalgia	0	0	0	0	0	1 (16.7)	1 (50.0)
Myalgia intercostal	0	0	0	0	0	0	1 (50.0)
***Gastrointestinal disorders***	**0**	**0**	**0**	**0**	**1 (12.5)**	**1 (16.7)**	**0**
Diarrhea	0	0	0	0	1 (12.5)	0	0
Dyspepsia	0	0	0	0	0	1 (16.7)	0
***Respiratory thoracic and mediastinal disorders***	**0**	**0**	**0**	**0**	**1 (12.5)**	**0**	**0**
Epistaxis	0	0	0	0	1 (12.5)	0	0
***Infections and infestations***	**0**	**1 (16.7)**	**0**	**0**	**0**	**0**	**0**
Nasopharyngitis	0	1 (16.7)	0	0	0	0	0

*System organ class* Preferred term, n (%)	EGT710 MAD (Part B)					
	Capsule				Suspension	
	**300 mg n = 6**	**460 mg n = 6**	**700 mg n = 5**	**Placebo n = 6**	**700 mg n = 6**	**Placebo n = 2**
**Participants with ≥1 AE**	**6 (100)**	**2 (33.3)**	**2 (40.0)**	**5 (83.3)**	**5 (83.3)**	**0**
***General disorders and administration site conditions***	**3 (50.0)**	**2 (33.3)**	**0**	**5 (83.3)**	**2 (33.3)**	**0**
Catheter site hematoma	0	0	0	0	1 (16.7)	0
Catheter site irritation	0	1 (16.7)	0	1 (16.7)	1 (16.7)	0
Medical device site irritation	2 (33.3)	2 (33.3)	0	5 (83.3)	1 (16.7)	0
***Gastrointestinal disorders***	**1 (16.7)**	**0**	**0**	**0**	**3 (50.0)**	**0**
Abdominal distension	0	0	0	0	3 (50.0)	0
Diarrhea	1 (16.7)	0	0	0	0	0
***Nervous system disorders***	**2 (33.3)**	**0**	**1 (20.0)**	**0**	**0**	**0**
Fatigue	2 (33.3)	0	0	1 (16.7)	0	0
Headache	1 (16.7)	0	1 (20.0)	0	0	0
***Respiratory thoracic and mediastinal disorders***	**2 (33.3)**	**0**	**0**	**0**	**1 (16.7)**	**0**
Cough	0	0	0	0	1 (16.7)	0
***Musculoskeletal and connective tissue disorders***	**0**	**0**	**1 (20.0)**	**0**	**1 (16.7)**	**0**
Muscle discomfort	0	0	0	0	1 (16.7)	0
Muscle spasms	0	0	1 (20.0)	0	0	0
***Infections and infestations***	**2 (33.3)**	**0**	**0**	**0**	**0**	**0**
COVID‐19	1 (16.7)	0	0	0	0	0
Nasopharyngitis	1 (16.7)	0	0	0	0	0
***Skin and subcutaneous tissue disorders***	**0**	**0**	**0**	**1 (16.7)**	**0**	**0**
Dry eye	1 (16.7)	0	0	0	0	0
Dry skin	0	0	0	1 (16.7)	0	0

*System organ class* Preferred term, n (%)	FE/rBA (Part C)		
	EGT710 600 mg		
	Capsule Fasted n = 18	Suspension Fasted n = 18	Suspension Fed n = 18
**Participants with ≥1 AE**	**6 (33.3)**	**5 (27.8)**	**7 (38.9)**
***General disorders and administration site conditions***	**3 (16.7)**	**2 (11.1)**	**5 (27.8)**
Medical device site irritation	0	0	1 (5.6)
Catheter site irritation	1 (5.6)	2 (11.1)	3 (16.7)
Fatigue	2 (11.1)	0	2 (11.1)
***Gastrointestinal disorders***	**3 (16.7)**	**3 (16.7)**	**3 (16.7)**
Abdominal discomfort	1 (5.6)	1 (5.6)	1 (5.6)
Diarrhea	2 (11.1)	2 (11.1)	2 (11.1)
Dyspepsia	1 (5.6)	0	0
Nausea	1 (5.6)	0	0
Oral pruritus	0	0	1 (5.6)
Aphthous ulcer	1 (5.6)	0	0
***Nervous system disorders***	**3 (16.7)**	**0**	**1 (5.6)**
Headache	2 (11.1)	0	1 (5.6)
Dizziness	1 (5.6)	0	0
***Psychiatric disorders***	**0**	**1 (5.6)**	**0**
Insomnia	0	1 (5.6)	0
***Musculoskeletal and connective tissue disorders***	**1 (5.6)**	**0**	**0**
Myalgia	1 (5.6)	0	0
***Infections and infestations***	**0**	**0**	**1 (5.6)**
Nasopharyngitis	0	0	1 (5.6)

AE, adverse event; FE/rBA, food effect/relative bioavailability; MAD, multiple ascending dose; SAD, single ascending dose.

The analysis set includes all participants randomized into the active treatment and placebo groups.

All AEs were Grade 1, with one case of catheter site irritation, in Part C, still recovering at the time of data cutoff (October 30, 2023; 57 days after the last participant last visit of September 03, 2023), while all other AEs had resolved. Investigators reported six AEs related to the study treatment: somnolence (n = 1) with EGT710 and loose stool (n = 1) with placebo in Part A; diarrhea (n = 1) and abdominal distension (n = 2) with EGT710 in Part B; oral pruritus (n = 1) with EGT710 suspension under fed state in Part C. The EGT710 suspension formulation was associated with a greater frequency of gastrointestinal adverse events 10 of the 30 (33.3%) participants compared with the EGT710 capsule formulation, 4 of the 58 participants (6.9%). No deaths or other serious AEs were reported during this study. No clinically relevant changes in clinical laboratory evaluations, vital signs, ECG parameters, or physical examinations were observed. Overall, there were no dose‐limiting or apparent dose‐dependent adverse effects.

### Pharmacokinetics Analysis

The PK analysis included 29 participants (capsule, n = 23; suspension, n = 6) in Part A, 23 participants (capsule, n = 17; suspension, n = 6) in Part B, and 18 participants in Part C. EGT710 was rapidly absorbed after oral administration, with maximum plasma concentration (C_max_) occurring typically within 2 h. The median T_max_ ranged from 1.52 to 2.00 h in Part A and 1.26 to 2.00 h in Part B at both Days 1 and 7. The EGT710 plasma concentration‐time profiles after single and multiple dose administration across the different doses appeared similar with no significant accumulation evident in Part B. C_max_ and AUC from the time of dosing to the last measurable concentration sampling (AUC_last_) increased sub‐proportionally with increasing doses of EGT710 (Figure 2 and Table 3). Apparent loss of exposure was not observed between Days 1 and 7 due to enzyme induction, and EGT710 exposure exhibited moderate inter‐individual variability with doses of 460 and 700 mg over 7 days. Geo‐mean total oral clearance (CL/F) of EGT710 ranged from 9110 to 14,700 mL/h, and the renal CL ranged from 0.025 to 0.051 L/h, indicating that EGT710 was mainly eliminated by non‐renal pathways (Table 3).

**Figure 2:**
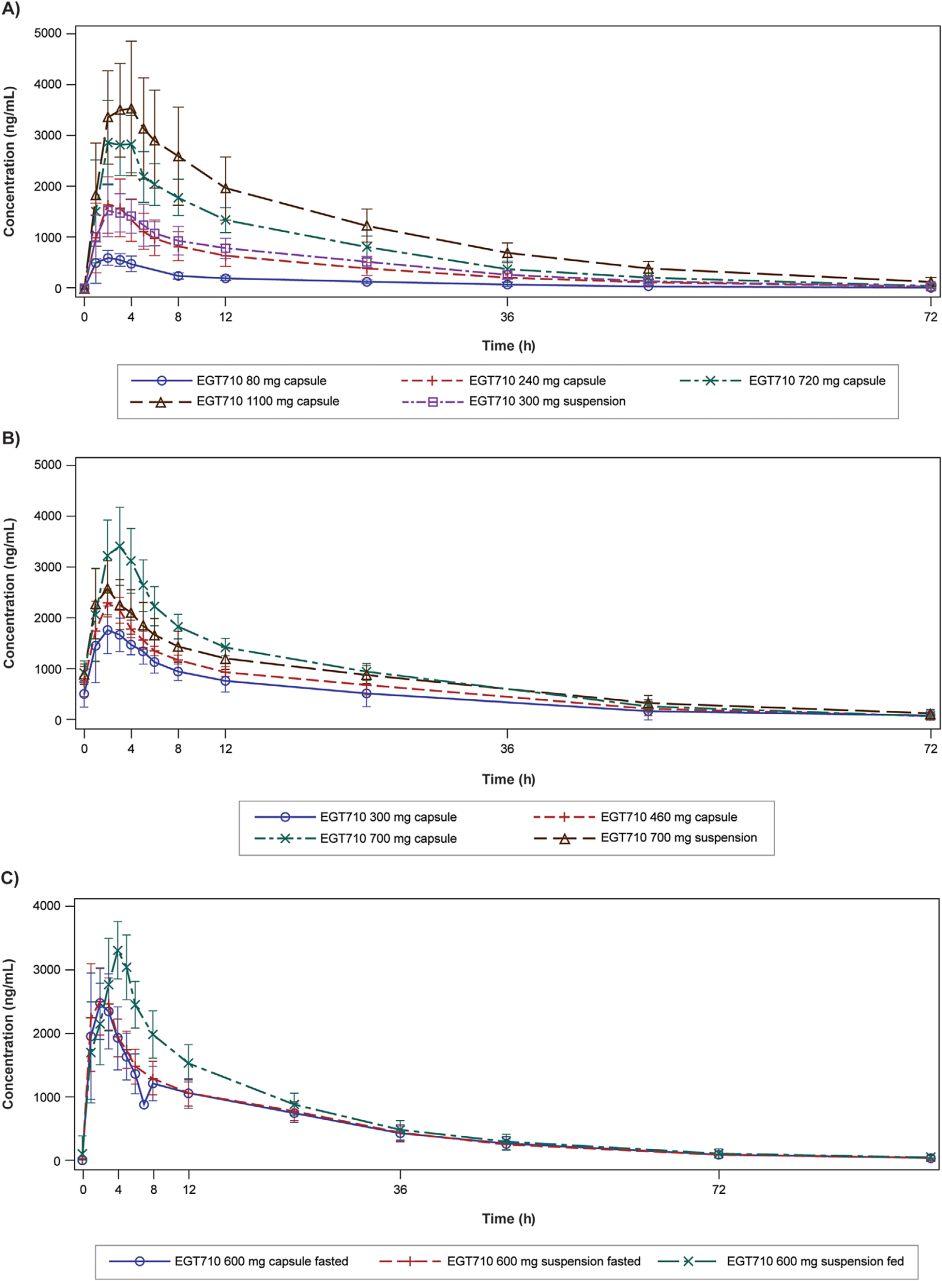
Mean (SD) plasma concentration–time profiles (linear view). (A) SAD, (B) MAD (Day 7), and (C) FE/rBA. FE/rBA, food effect/relative bioavailability; MAD, multiple ascending dose; SAD, single ascending dose.

**Table 3:** Summary of Plasma and Urine PK Parameters of EGT710

Parameter	EGT710 Single Dose				
	Capsule				Suspension
	80 mg n = 5	240 mg n = 6	720 mg n = 6	1100 mg n = 6	300 mg n = 6
**Plasma PK parameter**					
C_max_ (ng/mL)	714 (36.4)	1770 (26.6)	3110 (27.1)	3810 (29.9)	1650 (24.5)
T_max_ (h)	1.52 (0.50, 4.00)	2.00(1.00, 2.52)	2.00 (1.00, 4.00)	2.00 (1.02, 4.08)	2.00 (1.00, 5.00)
AUC_last_ (h ng/mL)	8380 (19.1)	24,200 (28.6)	48,300 (25.8)	72,000 (26.7)	28,600 (23.4)
AUC_last_/dose (h ng/mL/mg)	104.8	100.8	67.1	65.5	95.3
AUC_inf_ (h ng/mL)	8780 (20.3)	24,900 (28.7)	48,900 (26.5)	74,700 (27.1)	29,200 (23.8)
t_1/2_ (h)	15.1 (34.2)	13.7 (23.9)	10.7 (15.9)	13.8 (26.8)	12.3 (15.5)
CL/F (mL/h)	9110 (20.3)	9620 (28.7)	14,700 (26.5)	14,700 (27.1)	10,300 (23.8)
V/F (mL)	198,000 (31.4)	190,000 (35.7)	226,000 (13.8)	293,000 (36.0)	182,000 (21.9)
**Urine PK parameter**					
Amount excreted (ng)	152,000 (69.6)	726,000 (61.7)	1,780,000 (116.2)	2,460,000 (43.1)	888,000 (44.2)
Percentage excreted	0.190	0.302	0.247	0.224	0.296
Renal CL (L/h)	0.025 (76.7)	0.041 (50.6)	0.050 (96.3)	0.050 (29.2)	0.044 (32.6)

	**EGT710 Multiple Dose**			
	**Capsule**			**Suspension**
	**300 mg** **n = 6**	**460 mg** **n = 6**	**700 mg** **n = 5**	**700 mg** **n = 6**
**Plasma PK parameter**				
**Day 1**				
C_max_ (ng/mL)	1800 (26.8)	2280 (20.3)	3730 (20.9)	2520 (22.7)
T_max_ (h)	1.26 (1.00, 4.00)	1.50 (1.18, 2.02)	2.00 (1.50, 4.02)	1.76 (1.00, 4.00)
AUC_last_ (h ng/mL)	17,800 (15.8)	24,100 (12.2)	43,700 (19.2)	30,800 (25.2)
AUC_tau_ (h ng/mL)	17,900 (15.9)	24,300 (12.2)	43,900 (19.2)	30,900 (25.1)
AUC_0‐t24_ (h ng/mL)	17,900 (15.9)	24,300 (12.2)	43,900 (19.2)	30,900 (25.1)
CL/F_tau_ (mL/h)	16,800 (15.9)	19,000 (12.2)	15,900 (19.2)	22,600 (25.1)
V/F_tau_ (mL)	501,000 (50.6)	592,000 (60.9)	373,000 (34.2)	949,000 (89.0)
**Day 7**				
C_max_ (ng/mL)	1940 (26.7)	2490 (9.9)	3650 (19.8)	2680 (21.8)
T_max_ (h)	1.76 (1.00;4.00)	1.51 (1.02;2.48)	2.00 (1.50;4.00)	1.50 (1.50;2.00)
AUC_last_ (h ng/mL)	30,300 (40.8)	39,400 (21.7)	57,000 (15.3)	51,100 (25.1)
AUC_tau_ (h ng/mL)	21,300 (25.1)	27,100 (8.7)	40,900 (14.6)	33,100 (19.4)
AUC_inf_ (h ng/mL)	32,100 (48.1)	40,900 (25.4)	58,400 (16.1)	54,000 (27.8)
AUC_0‐t24_ (h ng/mL)	21,300 (25.1)	27,100 (8.7)	40,900 (14.6)	33,100 (19.4)
Accumulation ratio	1.19 (14.6)	1.12 (11.6)	0.931 (9.6)	1.07 (17.5)
CL/F_tau_ (mL/h)	14,100 (25.1)	17,000 (8.7)	17,100 (14.6)	21,100 (19.4)
V/F_tau_ (mL)	298,000 (30.3)	331,000 (20.2)	301,000 (39.4)	485,000 (22.5)
**Urine PK parameter (Day 7)**				
Amount excreted (ng)	1,510,000 (49.9)	1,260,000 (47.5)	1,640,000 (44.6)	2,620,000 (27.9)
Percentage excreted (%)	0.502 (49.9)	0.273 (47.5)	0.234 (44.6)	0.374 (27.9)
Renal CL (L/h)	0.05 (101.8)	0.032 (54.7)	0.029 (56.3)	0.051 (41.4)

	**EGT710 600 mg FE/rBA**		
	**Capsule Fasted** **n = 18**	**Suspension Fasted** **n = 18**	**Suspension Fed** **n = 18**
**Plasma PK parameter**			
C_max_ (ng/mL)	2830 (23.0)	2870 (19.1)	3430 (15.9)
T_max_ (h)	1.50 (0.50, 2.50)	2.0 (0.50, 5.00)	4.0 (0.50, 5.00)
AUC_inf_ (h ng/mL)	45,300 (17.1)	46,700 (16.2)	59,100 (17.8)
AUC_last_ (h ng/mL)	44,500 (16.5)	45,800 (15.4)	58,200 (16.6)
Cl/F (mL/h)	13,200 (17.1)	12,800 (16.2)	10,100 (17.8)
Vz/F (mL)	285,000 (24.7)	276,000 (23.3)	215,000 (18.9)

AUC_last_, AUC from time zero to the measurable concentration sampling; AUC_inf_, AUC from time zero to infinity; C_max_, mean maximum plasma concentration; CL/F_tau_, the total body clearance of the drug from the plasma; t_max_, median time to C_max_; t_1/2_, the elimination half‐life associated with the terminal slope (λz) of a semi‐logarithmic concentration–time curve (time); λz (lambda z), smallest (slowest) disposition (hybrid) rate constant (time^−1^) for terminal elimination; V/F_tau_, apparent volume of distribution during the terminal phase.

The analysis set includes all participants with at least one available valid PK concentration, who received any study treatment without any protocol deviations that impact PK data.

Data are expressed as Geo‐mean (CV%), except for T_max_, which is shown as median (range).

The oral bioavailability of EGT710 as suspension was similar to that of capsules at the dose level of 600 mg, with no clinically relevant differences in the time course of exposure. When taken with food, the exposure achieved was higher than the dosing without food (C_max_ [90% CI], 9%–31%; AUC [90% CI], 22%–32%). Fed/fasted ratios were 1.266 for AUC_inf_, 1.269 for AUC_last_, and 1.196 for C_max_. Summary statistics of plasma and urinary PK parameters are presented in Table 3.

### Effect on CYP3A4 and OAT1/3 Activity

An increase in the ratio of 6β‐hydroxycortisol to cortisol concentrations in urine from pre‐dose (Day −1 and Day 1) to post‐dose (Days 7, 8, 9, and 10) was noted for all participants in Part B (Figure 3). The post‐dose to pre‐dose ratio showed a dose‐dependent increase between 300 and 700 mg. For all cohorts, the mean pre‐dose ratio (5.77; SD:1.75) compared with the mean post‐dose ratio (13.44; SD: 3.61) revealed an induction ratio of 2.33, suggesting a weak‐to‐moderate CYP3A4 induction effect by EGT710 relative to the effect of index inducers on this biomarker.^15^

**Figure 3:**
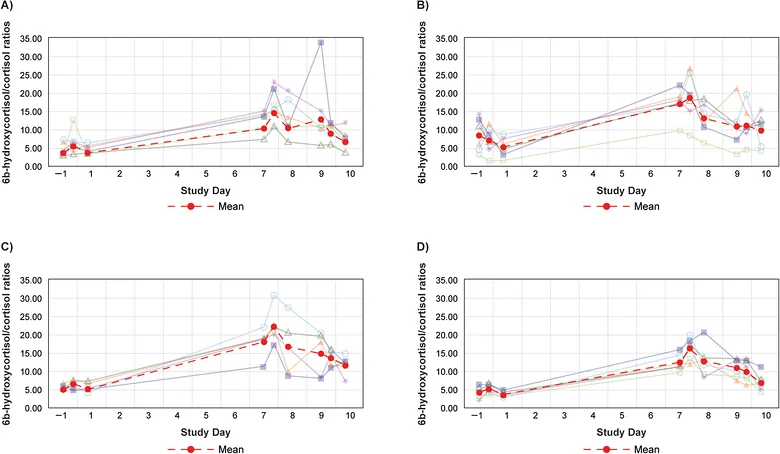
Effect of EGT710 on the CYP3A4 activity^a^. Individual and mean 6b‐OH‐C/C ratios for (A) 300 mg capsule cohort, (B) 460 mg capsule cohort, (C) 700 mg capsule cohort, and (D) 700 mg suspension cohort. ^a^Each semi‐transparent colored line in the graph represents an individual participant.

Evaluation of pre‐ and post‐dose plasma pyridoxic acid concentrations showed no clear trends, with a mean post‐/pre‐dose ratio of 0.93, suggesting that EGT710 may not affect OAT activity (Figure S2).

### Palatability

All 18 participants in Part C completed the questionnaire to assess taste, after taste, and smell of the suspension formulation under fasted and fed conditions. Most participants (14 [77.8%]) reported no noticeable smell. All participants who did notice a smell of the suspension (4 [22.2%]) described it as a “good” smell. Most participants reported a taste (fasted, 15 [83.3%]; fed state, 16 [88.9%]) and after taste (fasted, 14 [77.8%]; fed state, 13 [72.2%]). Of participants who noted a taste or after taste, the number of participants who described the taste as “bad” (fasted, 7 [38.9%]; fed state, 7 [38.9%]) and after taste as “bad” (fasted, 6 [33.3%]; fed state, 8 [44.4%]) was similar to those who described the taste as “good”(fasted, 6 [33.3%]; fed state 6 [33.3%]) and after taste as “good” (fasted, 8 [44.4%]; fed state, 5 [27.8%]).

## Discussion and Conclusions

Prevention and treatment of acute coronavirus disease are complementary strategies. Specific, safe and effective treatment remains a medical need. Despite the waning of the COVID‐19 pandemic, given vaccine hesitancy and continuous emergence of novel coronavirus variants that may evade pre‐existing immunity, development of safer, better tolerated, more efficacious broad‐spectrum antiviral treatments is prudent.

The three parts of this study were designed to answer key questions about potential future clinical use of EGT710 as a treatment for coronavirus disease (in addition to determining generally whether clinical development could or should continue). Collectively, the results of the three parts of the study preliminarily suggest that EGT710 should not require special management or special safety monitoring.

There were no dose‐dependent or dose‐limiting adverse effects in the study. Adverse events were generally mild in intensity (Grade 1) and occurred with a similar frequency between EGT710 and placebo groups, although the suspension formulation was associated with a higher incidence of gastrointestinal adverse effects than the capsule formulation. Generally, the safety and tolerability of EGT710 were acceptable and comparable to the published data for other coronavirus M^pro^ inhibitors.^18^, ^19^, ^20^

EGT710 was rapidly absorbed following oral administration. Despite the systemic exposure increasing in a less‐than‐proportional manner at the higher doses, putative therapeutic concentrations were achievable and the PK of EGT710 was predictable and able to be modeled. When the in vitro profiling results are considered, the effects on endogenous markers of CYP3A4 and OAT activity observed in this study suggest a low potential for clinically significant drug–drug interactions.^13^ By contrast, co‐administration of ritonavir, a PK enhancer and strong CYP3A4 inhibitor, is required to significantly increase nirmatrelvir exposure (ensuring effective concentrations). This, however, limits its use in patients taking concomitant CYP3A4‐sensitive medications.^20^, ^21^ The oral bioavailability of EGT710 when administered as a suspension was comparable to that of capsules, with no clinically relevant differences in the overall and time course of exposure. These results may establish the suspension as an option for treatment of patients who have feeding tubes or are unable to swallow capsules. Food modestly increased systemic exposure to EGT710 but not to an extent that is likely to require specific recommendations for administration with or without food.^22^

The duration of Part B in this study is directly relevant to the treatment of acute coronavirus disease with M^pro^ inhibitors. Therefore, the safety, tolerability, and PK data reflect the same timeframe (and range of exposures) for future investigation of the safety and efficacy of EGT710 in patients with acute coronavirus disease. The results of this study support proceeding to such an investigation.

The relatively small number of healthy adults enrolled from a single center, together with the predominantly male study population, may limit the generalizability of these findings to broader or different patient populations, including female patients, and those with varying medical complexities and concomitant treatments. It is possible that more severe or serious risks of EGT710 exist but were not observed in this study because of its size, duration of exposure, and/or its population. The results of this study suggest that treatment with EGT710 is likely to be manageable with minimal complications (e.g., drug–drug interactions, safety, and tolerability with concomitant drug therapies). However, the study duration of a maximum of 7 days may not have adequately characterized the maximum CYP3A4 induction effect of EGT710, which could be relevant for settings in which longer duration treatment of coronavirus disease may be necessary. The results of this first‐in‐human study do not answer all the practical, clinical questions. Therefore, clarifying the therapeutic utility and the optimal clinical use of EGT710 requires additional investigation in patients. Overall, the results of this study provide the rationale for continued clinical investigation to establish the safety and efficacy of EGT710 as a potential treatment for acute coronavirus disease.

## Author Contributions

Jart A. F. Oosterhaven contributed to the investigation, resources, conceptualization, study design, methodology, execution, interpretation of the study, and supervision. Jonathan Trolander, Lidiya Bebrevska, Hilmar Schiller, Begum Alaybeyoglu, Suzanne Gaudet, Allison Claas, Kamal Kumar Balavenkatraman, Renee Bergeron, Fernanda V. Duraes, Swati Ghanshani, Elisabete Goncalves, Frederique Chaperon, and Steven J. Kovacs contributed to conceptualization, methodology, supervision, funding acquisition, project administration, study design, execution, analysis, and interpretation of the study. All authors critically reviewed and revised the manuscript and approved the final version for submission. All authors made critical contributions to the conception, design, data interpretation, drafting, and revision of the manuscript. All authors approved the final version of the manuscript and agreed to be accountable for all aspects of the work ensuring integrity and accuracy. All authors had direct access to and verified the underlying data reported in the manuscript.

## Conflicts of Interest

Jart A. F. Oosterhaven works as a research physician at ICON Plc, a contract research organization (CRO) that was contracted by Novartis to conduct this study. Fernanda V. Duraes was an employee of Novartis Pharma AG, Basel, Switzerland during the time of the study, and holds stocks. She is a current employee of Roche, Basel, Switzerland; Jonathan Trolander, Lidiya Bebrevska, Hilmar Schiller, Begum Alaybeyoglu, Suzanne Gaudet, Allison Claas, Kamal Kumar Balavenkatraman, Renee Bergeron, Frederique Chaperon, Swati Ghanshani, Elisabete Goncalves, and Steven J. Kovacs are full‐time employees of Novartis and are stockholders or eligible to hold stocks with the company.

## Funding

Novartis Pharma AG, Basel, Switzerland, funded this study, with support, in whole or in part, by the Bill & Melinda Gates Foundation.

## Data Sharing

Novartis is committed to sharing with qualified external researchers, access to patient‐level data and supporting clinical documents from eligible studies. These requests are reviewed and approved by an independent review panel on the basis of scientific merit. All data provided are anonymized to respect the privacy of patients who have participated in the trial in line with applicable laws and regulations. The availability of the study data is according to the criteria and process described on www.clinicalstudydatarequest.com

## Previous Data Presentation Information

Figures 1 and 2 have been presented twice, once as a poster at The American Society of Tropical Medicine and Hygiene (ASTMH) conference on November 16, 2024, and in another poster at the Figon Dutch Medicine Days October 9, 2025 by same authors.

## Trial Registration ID

EudraCT 2022‐001905‐37‐NL.

## Supporting information



Supporting information

Supporting information

Supporting information
