# Supplementary material for: Safety, Tolerability, and Pharmacokinetics of EGT710, a Novel Coronavirus Mpro Inhibitor, in Healthy Adults: A Randomized, Single‐ and Multiple‐Dose Study
Source: Clin Pharmacol Drug Dev. 2026 Oct 24;15(10):e70115. doi: 10.1002/cpdd.70115 (PMC13695357; doi:10.1002/cpdd.70115)
Supplement: Supplementary file 1 [file CPDD-15-0-s003.docx]

**Supplementary Figure Titles and Legends**

**Figure S1. Trial profile**

**Legend:** FE/rBA, food effect/relative bioavailability; MAD, multiple ascending dose; SAD, single ascending dose.

**Figure S2. Effect of EGT710 on OAT activity**

**Legend:** ^a^Pyridoxic acid post-/pre-dose ratio per participant.
